# A Blast From the Past: Toxoplasmic Encephalitis As the Initial Presentation of HIV/AIDS

**DOI:** 10.7759/cureus.58693

**Published:** 2024-04-21

**Authors:** Olivia R Ortiz, Tara Norris

**Affiliations:** 1 Internal Medicine, Methodist Health System, Dallas, USA

**Keywords:** toxoplasmic encephalitis, post-art era, immunocompromised, brain mass, hiv aids, cerebral toxoplasmosis

## Abstract

Many opportunistic infections (OIs) seen early in the human immunodeficiency virus (HIV) epidemic receded in prevalence with the advent of antiretroviral therapy (ART). Despite the availability of early detection and treatment of HIV as well as guidelines for near-universal screening, there remains a sizable population of individuals living with HIV who are not yet aware of their HIV status. These individuals are at risk for OIs such as toxoplasmosis, which would otherwise be preventable with ART and appropriate prophylaxis. Toxoplasmic encephalitis (TE) usually occurs in the late stages of HIV with acquired immunodeficiency syndrome (AIDS), but we present a case of a 38-year-old female with TE as the initial presentation of HIV/AIDS. Testing for the presence of an immunocompromising condition such as HIV is important in patients presenting with focal brain lesions as the differential diagnosis will change, and proper workup may spare invasive procedures such as a brain biopsy.

## Introduction

*Toxoplasma gondii* is an obligate intracellular opportunistic parasite that can infect human hosts via contact with or ingestion of oocytes shed in feline feces, consumption of undercooked meat containing tissue cysts, or vertical transmission from mother to fetus [1,2]. *T. gondii* is a relatively common pathogen, with an estimated 11% of the United States (US) population over the age of six years having been infected [3]. Infection rates are up to 60% of the population in some parts of the world, particularly in hot, humid climates and lower altitudes, which promote improved oocyst survival. *T. gondii* causes toxoplasmosis, which is generally asymptomatic in immunocompetent hosts, but may manifest as a mild flu-like illness in rare cases. *T. gondii* can persist in the body for many years and may become reactivated and cause more serious disease if the host becomes immunocompromised [2,4].

Toxoplasmic encephalitis (TE) was the most common cause of focal brain lesions in patients with human immunodeficiency virus (HIV) and acquired immunodeficiency syndrome (AIDS) in the era before antiretroviral therapy (ART), and even with the introduction of ART therapy, it still remains one of the top causes of focal brain lesions in the HIV/AIDS population [5,6]. TE can present with solitary or multiple ring-enhancing lesions with mass effect, often found in the frontal and parietal lobes, corticomedullary junction, and basal ganglia [5]. Common clinical features of infection include fever, headache, altered mental status, seizures, and focal neurological deficits [7]. A TE diagnosis is most commonly made through serologic testing in the setting of suggestive radiologic findings and clinical symptoms, but parasites may also be directly observed on stained biopsy material or cerebrospinal fluid (CSF) if the diagnosis is in question [4]. For patients with classic imaging findings who are *T. gondii *seropositive, have clusters of differentiation 4 (CD4) count <200 cells/μL, and have not been on TE prophylaxis, empiric treatment for TE with close follow-up is a reasonable management strategy that avoids more invasive testing such as brain biopsy [1,8]. A joint guideline from the Centers for Disease Control and Prevention (CDC), National Institutes of Health, and the HIV Medicine Association of the Infectious Disease Society of America recommends testing for *Toxoplasma* IgG soon after the diagnosis of HIV to detect latent infection, initiating counseling and education over sources of *Toxoplasma* infection, and starting anti-*Toxoplasma* prophylaxis in seropositive patients with CD4 counts <100 cells/μL [2]. We present a case of a 38-year-old female with TE as the initial presentation of HIV/AIDS.

## Case presentation

A previously healthy 38-year-old female presented to an outside emergency department with five days of a left parietal headache. She described the headache as constant, of moderate intensity, and throbbing in quality. The headache was associated with photophobia, nausea with vomiting, and was incompletely relieved with acetaminophen. On review of systems, the patient also reported generalized weakness and loss of appetite and described several brief episodes of expressive aphasia in the two months prior to presentation. She denied fever, chills, shortness of breath, cough, dizziness, loss of consciousness, or numbness. The patient had no known past medical or surgical history. She was born in Cameroon and moved to the US nine years prior. At the outside hospital, the patient was afebrile and hemodynamically stable. A physical exam was positive only for mild weakness in the right upper and lower extremities compared to the left. Computed tomography (CT) of the head without contrast showed multifocal areas of vasogenic edema in the left frontoparietal lobe, left temporal lobe, and right occipitoparietal lobe, the largest of which measured 7 x 5 cm. A left-to-right midline shift of 5 mm was also noted. The radiology report said these findings were concerning for metastatic disease. Dexamethasone and levetiracetam were started for cerebral edema and seizure prophylaxis, respectively.

**Figure 1:**
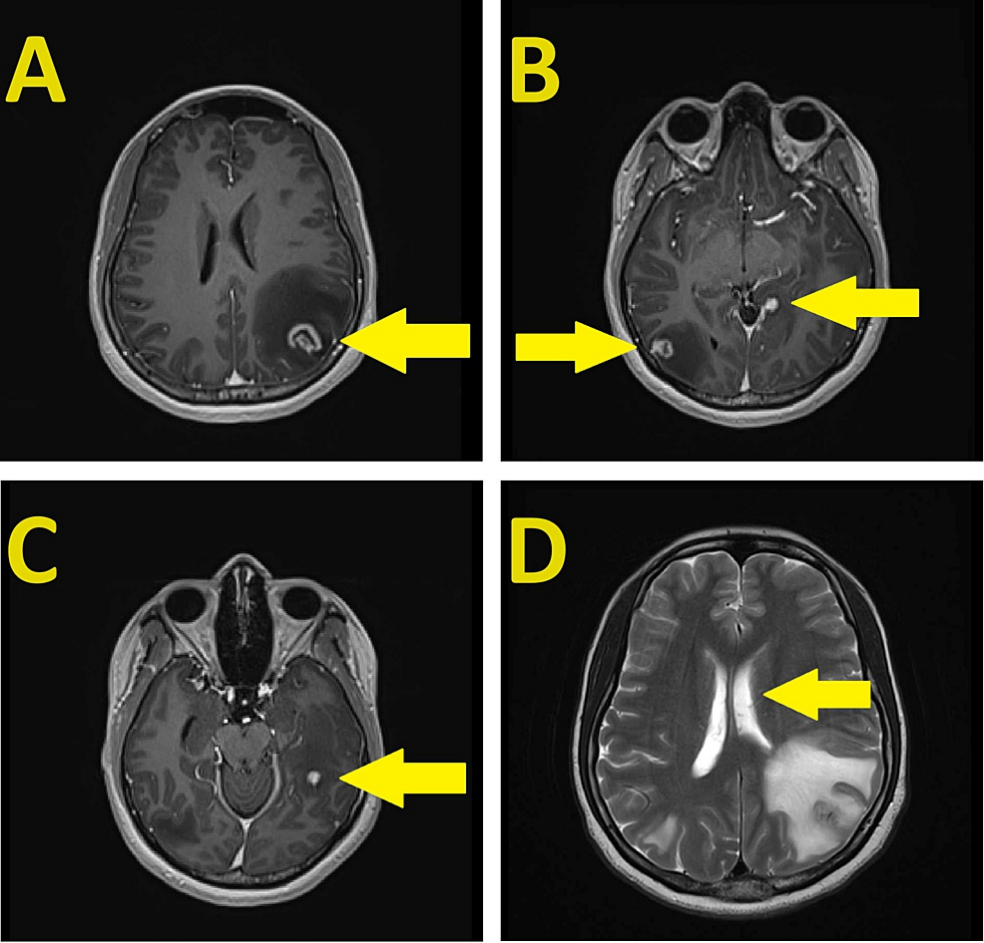
Magnetic resonance imaging (MRI) scans of the brain (A-C) Post-contrast enhanced MRI axial T1-weighted stealth sequences showing four contrast-enhancing lesions surrounded by vasogenic edema. The lesions are located in the left parietal lobe (A), right occipital lobe and left occipital lobe (B), and left temporal lobe (C). The largest lesion measures 1.65 x 1.79 cm (A). (D) An axial T2 MRI image showing a 4-5 mm left-to-right midline shift of the patient.

The patient was subsequently transferred to our facility for a higher level of care, and she was admitted to the Neurological Critical Care unit. Magnetic resonance imaging (MRI) of the brain performed with and without contrast showed numerous abnormal enhancing parenchymal masses within the bilateral cerebral hemispheres surrounded by vasogenic edema, the largest of which was a heterogeneous enhancing mass of 1.6 x 1.7 x 1.5 cm in the left parietal region (Figures 1A-1C). A left-to-right midline shift of 4-5 mm was again demonstrated (Figure 1D). CT of the chest/abdomen/pelvis showed no evidence of malignancy. A stereotactic brain biopsy of the left parietal mass was pursued due to the brain lesions being of undetermined etiology. A frozen section came back consistent with a possible infectious etiology. The patient was started on empiric broad-spectrum antibiotics and remained on steroids and seizure prophylaxis.

An infectious workup was then performed, and the patient was found to be HIV positive with a CD4 count of 54 cells/μL. *T. gondii* IgM was negative, but IgG was strongly positive at 320 IU/mL. Pathology from the brain biopsy subsequently revealed a *T. gondii* infection with necrosis and gliosis. The patient was treated with intravenous trimethoprim-sulfamethoxazole (TMP-SMX) during her stay. She was incidentally found to have both a positive COVID-19 antigen and polymerase chain reaction (PCR) but displayed no symptoms throughout her hospital course, so this was considered noncontributory. The patient’s neurologic status improved, and she was discharged home on pyrimethamine, leucovorin, and sulfadiazine for six weeks, along with levetiracetam for seizure prophylaxis. Antiretroviral therapy was to be initiated upon outpatient follow-up after at least two weeks of antimicrobial treatment.

Following discharge, the patient was unable to obtain the sulfadiazine due to difficulties with her pharmacy. TMP-SMX was prescribed at her infectious disease follow-up appointment as an alternative agent. She had no documented sulfa allergy and had tolerated the drug well in the hospital, but within several hours of the first dose of oral TMP-SMX, she developed a diffuse macular rash with associated pruritus and shortness of breath. She presented to the emergency department, where she was diagnosed with an allergic reaction to the sulfa drug and was readmitted for further management. CT of the brain at the time of readmission (approximately four weeks after initial presentation) showed near-complete resolution of the vasogenic edema seen on previous imaging, indicating a positive response to the treatment. She was recommended by the infectious disease specialty to undergo desensitization to sulfa, given the pivotal role that these drugs play in the treatment of TE. The desensitization protocol was successful and the patient was discharged home on TMP-SMX alone. The final duration of therapy was to be determined based on continued clinical response.

## Discussion

Our patient’s presentation of multiple contrast-enhancing brain lesions was a diagnostic conundrum because her immunocompromised state was unknown. In immunocompromised patients, the differential diagnosis of focal brain lesions expands to include numerous infectious sources in addition to primary malignancy or metastatic disease. The most common causes of ring-enhancing lesions in immunocompromised patients include TE, primary central nervous system lymphoma, tuberculoma, Cryptococcus infection, metastatic or primary malignancy, and bacterial or fungal abscesses [7]. As it was initially unknown that our patient was immunocompromised, TE was lower on the differential, and thus the diagnosis was delayed. An anchoring bias initially appeared to be at play when the focus was on evaluation for malignancy with a presumptive diagnosis of metastatic disease. This bias delayed diagnosis of an immunocompromised state, initiation of antibiotics, and resulted in the patient undergoing potentially unnecessary non-invasive and highly invasive testing. A detailed social history revealed that our patient hailed from Sub-Saharan Africa, a location with a large burden of HIV infection, and in those infected, a high coinfection rate with *T. gondii* [9]. While reports of TE as the initial presentation of HIV/AIDS are rare, the majority of such cases in the literature have been in individuals of non-US origin, with three having immigrated from Sub-Saharan Africa like our patient [10-13]. Given our patient’s history and imaging findings, earlier HIV testing and subsequent *T. gondii* testing would have allowed for earlier diagnosis, empiric treatment for TE, and avoidance of an invasive biopsy. This case serves as a reminder to keep the differential diagnosis of focal brain lesions wide and to consider all potential risk factors for disease that can aid in timely work-up and diagnosis.

In regards to treatment, TE can be life-threatening if not treated with appropriate antimicrobials [1]. Initial therapy is a combination of pyrimethamine, sulfadiazine, and leucovorin. If patients are unable to take sulfadiazine, clindamycin can be substituted. If pyrimethamine is unable to be used or obtained, TMP-SMX monotherapy can be used. Patients with a sulfa allergy should undergo sulfa desensitization, as in our case [2]. After TE treatment has been initiated, the patient should have clinical and imaging improvement in 2-3 weeks, and repeat head imaging is recommended to monitor for improvement of infection, specifically looking for a reduction in the size of the lesions, surrounding edema, and contrast enhancement [8]. Treatment should be continued for at least six weeks if there is radiologic and/or clinical improvement, after which chronic maintenance therapy should be employed for at least six months until the CD4 count is >200 cells/μL [2].

In patients with HIV, primary prophylaxis for TE is initiated at CD4 counts <100 cells/μL and can overlap with *Pneumocystis jiroveci* pneumonia (PJP) prophylaxis, which is initiated in patients with CD4 counts <200 cells/μL. Daily double-strength TMP-SMX, a first-line treatment for TE prophylaxis, also covers PJP prophylaxis. Atovaquone monotherapy can be used for TE prophylaxis, or if a patient is receiving dapsone for PJP prophylaxis, pyrimethamine plus leucovorin can be added. TE prophylaxis can be stopped after CD4 counts have increased to >200 cells/μL for more than three months [2].

The incidence of TE has decreased since the introduction of ART [14,15]. Studies examining the post-ART era have demonstrated decreased *Toxoplasma*-related hospitalizations in the HIV population, decreased cases of TE, and lower mortality risk in patients with TE [14-16]. In a study comparing the pre- and post-ART era focal brain lesions in the AIDS population, TE prophylaxis was associated with decreased risk for TE [17]. Following the initial decrease in the incidence of TE attributable to ART, its incidence has remained steady in recent years [14,18].

There are several proposed explanations for the sustained rate of TE among people living with HIV/AIDS (PLWHA) since the late 1990’s. First, about 13% of the >1.1 million PLWHA in the US are undiagnosed, just like our patient [19]. Delayed HIV diagnosis places patients at risk for presenting at a more advanced stage of immune dysfunction and infection due to lack of treatment with ART and appropriate opportunistic infection (OI) prophylaxis. Despite the institution of HIV screening guidelines for all patients ages 15 to 65 years old, median CD4 counts at HIV diagnosis have remained stable since 2009, suggesting many patients are still receiving a delayed diagnosis [20]. Secondly, noncompliance with ART, TE prophylaxis, and general loss to follow-up remain significant concerns despite improved access to care, early initiation of ART, and OI prophylaxis in patients newly diagnosed with HIV [20]. According to the CDC’s HIV statistics, 42 out of every 100 patients with new HIV diagnoses were lost to care, and thus are at higher risk of OIs [19]. Mayor et al. demonstrated that patients with TE in the post-ART era still presented with a high degree of immune dysfunction despite having been prescribed ART and TE prophylaxis, suggesting noncompliance as a significant contributing factor [15]. Thirdly, disparities in education and income contribute to higher seroprevalence in certain populations despite an overall decrease in seroprevalence of *T. gondii* in the US [3]. Higher seroprevalence in other regions of the world is putatively due to the above-noted factors as well as differences in access to safe drinking water, sanitization, cooking habits, meat preservation, and climate [9].

## Conclusions

*T. gondii* infection is relatively common and has the potential to cause life-threatening neurologic disease in immunocompromised hosts. While the prevalence of TE decreased demonstrably in the post-ART era, rates have since stabilized due to a variety of factors, including delayed HIV testing and diagnosis that can allow patients to present with late-stage OIs. An immunocompromised state should be ruled out in patients presenting with ring-enhancing lesions, and TE should remain high on the differential diagnosis, particularly when the CD4 count is <200 cells/μL. In the general population, screening for HIV followed by appropriate testing for OIs such as *T. gondii* and initiation of appropriate prophylaxis are key components in the prevention of significant disease with reactivation of prior infection.
